# Skeletal muscle–heart crosstalk in chronic kidney disease: hierarchical signaling networks underlying myocardial metabolic reprogramming and fibrotic remodeling

**DOI:** 10.3389/fphys.2026.1886745

**Published:** 2026-08-14

**Authors:** Haijing Lu, Boyin Liu, Jiazhou Ji, Lin Shuai, Jingyuan Cao

**Affiliations:** 1 Department of Nephrology, The Affiliated Taizhou People’s Hospital of Nanjing Medical University, Taizhou School of Clinical Medicine, Nanjing Medical University, Taizhou, Jiangsu, China; 2 Medical School of Nantong University, Nantong, China; 3 Medical School of Southeast University, Nanjing, China

**Keywords:** chronic kidney disease, extracellular vesicles, metabolic reprogramming, myocardial fibrosis, myokines, skeletal muscle–heart crosstalk

## Abstract

Cardiovascular disease is a major complication of chronic kidney disease (CKD) and often develops alongside skeletal muscle wasting and sarcopenia. These abnormalities are usually studied as separate consequences of CKD, but they may also be linked through shared systemic stressors and inter-organ communication. CKD exposes both skeletal muscle and the myocardium to a persistent uremic milieu characterized by toxin retention, chronic inflammation, oxidative stress, hypoxia, and metabolic disturbance. In this setting, skeletal muscle–heart crosstalk may shift from an adaptive homeostatic program to a maladaptive network that contributes to myocardial metabolic dysfunction and fibrotic remodeling. Under physiological conditions, and especially during exercise, skeletal muscle releases myokines and extracellular vesicles carrying miRNAs, proteins, and other regulatory molecules that support myocardial substrate utilization, mitochondrial function, and repair responses. In CKD, however, altered myokine profiles and dysregulated extracellular vesicle cargoes may act on cardiomyocytes, cardiac fibroblasts, and endothelial cells, promoting impaired metabolic flexibility, extracellular matrix deposition, and progressive cardiac remodeling. The heart may also feed back on skeletal muscle through cardiac-derived endocrine signals and neurohumoral pathways, further reinforcing muscle wasting and systemic dysfunction. In this review, we summarize current evidence on skeletal muscle–heart communication under physiological, exercise-related, and CKD-associated conditions, with emphasis on myokines, extracellular vesicles, and miRNA-mediated signaling. Better definition of this axis may help identify biomarkers and therapeutic targets for CKD-associated sarcopenia and cardiovascular disease.

## 1 Background

Muscle is central to locomotion and systemic metabolic homeostasis. Traditionally, it has been regarded as an effector tissue responsible for contraction and relaxation. Since the 1990s, however, skeletal muscle has increasingly been recognized as an active endocrine organ (Pedersen and Febbraio, 2012). In 1994, Ullum et al. observed a marked increase in circulating interleukin-6 (IL-6) during exercise in humans, providing early evidence that contracting skeletal muscle can influence distant organs through humoral signaling (Ullum et al., 1994). Subsequent studies have shown that skeletal muscle secretes a broad range of bioactive mediators, including myokines, metabolism-related factors, and extracellular vesicles (EVs), which regulate metabolic and inflammatory responses in the brain, heart, liver, gut, and other organs (Pedersen and Febbraio, 2012; Severinsen and Pedersen, 2020). These findings established skeletal muscle as an important source of inter-organ signals in addition to its mechanical function.

Beyond its canonical mechanical role as a pump, the myocardium functions as a specialized endocrine organ. Atrial and ventricular cardiomyocytes synthesize and release natriuretic peptides, which regulate fluid balance, vascular tone, and neurohumoral homeostasis (Burnett, 2019). In 1997, McPherron, Lawler, and Lee identified myostatin as a key negative regulator of skeletal muscle mass through genetic studies (McPherron et al., 1997). Myostatin was subsequently shown to be expressed in the myocardium and upregulated under pathological cardiac stress. Once released into the circulation, cardiac-derived myostatin can suppress skeletal muscle growth (Breitbart et al., 2011). Thus, skeletal muscle and the heart do not operate in isolation but communicate through muscle- and cardiac-derived factors, EVs, and neural, neurohumoral, and hemodynamic pathways.

Chronic kidney disease (CKD) is not confined to progressive renal dysfunction; it also creates a systemic pathophysiological milieu characterized by uremic toxin retention, chronic inflammation, oxidative stress, hypoxia, and metabolic imbalance. Clinical observations are consistent with functional coupling between skeletal muscle and the heart. In patients with advanced heart failure, the severity of skeletal muscle atrophy is not always fully proportional to reductions in physical activity (Toth et al., 2010; Callahan and Toth, 2013), whereas cardiomyopathy is common in inherited muscle disorders such as Duchenne muscular dystrophy and has important prognostic consequences (Kamdar and Garry, 2016; Cheeran et al., 2017). In CKD and dialysis populations, muscle wasting and cardiovascular structural abnormalities frequently coexist. Our group has reported associations of lower skeletal muscle mass and density with thoracic aortic calcification progression and cardiac valvular calcification (Li et al., 2025; Wang XX. et al., 2025). Experimental studies of CKD-associated sarcopenia and uremic cardiomyopathy further implicate EVs and their miRNA cargoes in cross-tissue signal transfer, providing a rationale for mechanistic investigation of skeletal muscle–heart communication in CKD.

Despite growing interest in skeletal muscle–heart communication, most studies have examined individual mediators or single-organ outcomes. How CKD reorganizes this communication as an integrated system remains poorly understood. In this review, we first summarize skeletal muscle–heart crosstalk under physiological conditions and during exercise and then examine how the uremic milieu shifts this communication toward maladaptive remodeling. We focus on myokines, EVs, and miRNA cargoes as potential links between CKD-associated muscle wasting, myocardial metabolic dysfunction, and fibrotic remodeling. To facilitate critical appraisal, we use five non-hierarchical and potentially overlapping evidence descriptors throughout the review: source-specific inter-organ evidence, recipient-organ or recipient-cell mechanistic evidence, exogenous or engineered-delivery evidence, human physiological or interventional evidence, and human observational evidence. **Table 1** summarizes representative preclinical and mechanistic studies, whereas **Table 2** summarizes selected human physiological, interventional, and observational studies. These descriptors define experimental context and inferential boundaries rather than constituting a formal quality-grading system.

**Table 1:** Exercise-associated muscle-derived mediators implicated in skeletal muscle–heart crosstalk.

Mediator	Key determinants	Receptor	Core pathways	Cardiac targets	Key effects
IL-6	Exercise modality (aerobic and/or resistance); intensity and duration; recruited muscle mass	IL-6R, gp130	JAK–STAT3; MAPK; PI3K-AKT; AMPK	CM, EC, VSMC, Imm	Promotes substrate mobilization and redistribution; enhances glucose uptake and fatty acid oxidation; induces anti-inflammatory cascades (e.g., IL-10, sTNF-R) and restrains excessive inflammation; overall supports post-exercise recovery and adaptation (whereas chronically sustained elevation may be associated with maladaptive changes)
Irisin	Aerobic, resistance, and HIIT; strongly influenced by exercise prescription, participant characteristics, and assay methodology	integrin αV-containing complexes (e.g., αVβ5/αVβ1)	p38/ERK; PI3K–AKT; AMPK–PGC-1α	CM, EC	Supports mitochondrial homeostasis and stress resilience; facilitates metabolic adaptation; reported in some models to be associated with anti-apoptotic, anti-oxidative, and anti-fibrotic profiles
Apelin	Exercise-induced apelin expression; linked to metabolic load	APJ	PI3K–AKT; AMPK; ERK1/2; eNOS	CM, EC, VSMC, CF	Facilitates vasodilation and perfusion and exerts pro-angiogenic effects; in selected models, apelin signaling has been associated with attenuation of remodeling and fibrosis under pathological stress
Musclin (Osteocrin, OSTN)	Upregulated with exercise; also influenced by energy status and environmental temperature	NPR-C	NP–cGMP–PKG	CM, CF, EC, VSMC	Helps maintain or improve systolic/diastolic signaling; suppresses fibroblast activation and collagen deposition; suggested protective effects in pressure-overload or HFpEF-related phenotypes
Myostatin–Follistatin	More closely linked to resistance training and muscle anabolic/catabolic balance	ActRIIA/ActRIIB (myostatin); follistatin acts as a ligand trap	Smad2/3	Indirect/systemic	Regulates the balance between muscle anabolism and catabolism and may shape a systemic milieu permissive for metabolic adaptation; direct causal contributions to myocardial remodeling remain incompletely defined
FSTL1	Resistance exercise-associated; increased skeletal muscle expression with release into circulation	DIP2A	PI3K–AKT–mTOR; Bcl-2/Bax	CM, EC, CF	Mediates anti-apoptotic and pro-repair effects; promotes angiogenesis and perfusion recovery; attenuates fibrosis and collagen deposition and ameliorates metabolic burden (in selected studies)
METRNL	More frequently reported with HIIT	Receptor not clearly defined	AMPK–HDAC4–GLUT4	CM	Increasing evidence links METRNL to improved metabolic phenotypes and/or inflammatory milieu; current evidence more strongly suggests involvement in exercise-related cardiac adaptation rather than establishing a definitive causal chain
Other exercise-responsive factors (e.g., BDNF, IGF-1)	Highly responsive to exercise; substantial variability across protocols and populations	BDNF: TrkB; IGF-1: IGF-1R	BDNF: PI3K–AKT, MAPK; IGF-1: PI3K–AKT–mTOR	CM, EC	Supports repair and adaptation; complementary pathways contributing to muscle–heart communication

CM, cardiomyocytes; EC, endothelial cells; VSMC, vascular smooth muscle cells; CF, cardiac fibroblasts; Imm, immune cells; HFpEF, heart failure with preserved ejection fraction; HIIT, high-intensity interval training.

**Table 2:** Design characteristics and interpretive boundaries of human studies informing skeletal muscle–cardiovascular relationships under physiological, exercise-related, and CKD-associated conditions.

Panel A. Human physiological, exercise, and interventional studies							
Reference, study design, and evidence descriptor	Population and sample characteristics	Clinical or physiological context	Exposure/intervention and comparator	Key assessments	Follow-up, attrition, and exclusions	Statistical adjustment and multiplicity	Main finding and interpretive boundary
Ullum et al., 1994; acute within-participant study; human physiological or interventional evidence	17 healthy, moderately trained young men; other demographics NR	Healthy participants; acute exercise physiology	1-h cycling at 75% VO_2_max; serial pre-, during-, and post-exercise sampling; no non-exercise control	Plasma IL-6, IL-1α, IL-1β, TNF-α; BMNC cytokine pre-mRNA; monocyte phenotypes; no cardiac endpoint	Acute sampling; dropout N/A; exclusions NR	Within-participant comparisons; covariate adjustment N/A; multiplicity NR	Plasma IL-6 increased during exercise without increased BMNC cytokine pre-mRNA. Skeletal-muscle origin and cardiac target engagement were not established; male-only acute design limits generalizability.
Engeli et al., 2012; pre–post training study; human physiological or interventional evidence	18 adults with obesity enrolled; 10 underwent the 8-week training component	Obesity; exercise adaptation; no CKD	Eight-week aerobic training; within-participant pre/post comparison; no parallel control	Vastus lateralis NPRA/GC-A, PGC1A, OXPHOS markers, mtDNA; VO_2_max, resting metabolic rate, ANP/BNP	Eight weeks; 10/18 participated; reasons for non-participation NR	Paired analyses; pathway-level adjustment for transcriptomic analyses; no clinical covariate adjustment	Training increased NPRA, PGC1A, OXPHOS-related measures, VO_2_max, and resting metabolic rate without increasing resting ANP/BNP. Causation by cardiac natriuretic peptide release was not demonstrated
Ji et al., 2024; randomized crossover exercise study; human physiological or interventional evidence	Nine men; age 24.0 ± 0.4 years; BMI 25.2 ± 0.7 kg/m²	Healthy participants; acute exercise physiology	No-exercise control, MICE, VICE, and HIIE sessions separated by 1 week	Serial serum FGF-21, FSTL1, cathepsin B, BDNF, and lactate; no cardiac endpoint	Sampling to 90 min post-exercise; attrition NR	Repeated-measures comparisons; covariate adjustment N/A; multiplicity NR	HIIE produced the largest acute biomarker responses. The small male-only study did not establish muscle origin, inter-organ transfer, or cardiovascular effects.
Whitham et al., 2018; acute exercise study; human physiological or interventional evidence	11 healthy men	Healthy participants; graded endurance exercise	1-h cycling at 55%, 70%, and 80% VO_2_max; rest, post-exercise, and 4-h recovery sampling	Plasma EV proteomics; leg arteriovenous protein flux; no direct cardiac uptake or phenotype	Acute study with 4-h recovery; dropout N/A; exclusions NR	Proteomic filtering and flux analyses; clinical covariate adjustment N/A	Exercise altered EV-associated proteins and identified candidate limb-released proteins. Arteriovenous flux did not establish skeletal-muscle-cell origin or EV delivery to the heart.
Frühbeis et al., 2015; pilot acute exercise study; human physiological or interventional evidence	12 healthy, physically active men: cycling cohort n=8, age 41.1 ± 14.9 years; running cohort n=4, age 27.0 ± 2.1 years	Healthy participants; acute exercise physiology	Incremental cycling to exhaustion; additional running observations; pre-, post-, and 90-min recovery sampling.	Circulating small-EV abundance and markers; lactate and cfDNA; no cardiac endpoint	Sampling to 90 min; dropout N/A; exclusions NR	Within-participant comparisons; covariate adjustment N/A; multiplicity NR	Circulating small EVs increased rapidly and declined during recovery. Cellular origin, cargo function, recipient organ, and cardiovascular relevance were not determined; the small male-only sample and early EV methods limit generalizability.
Wang et al., 2024; comparative exercise-intervention component of a mixed translational study; human physiological or interventional evidence	100 patients with HFmrEF (LVEF 40%–49%; NYHA II–III); 50 allocated to MICT and 50 to HIIT; detailed demographics NR	Heart failure; non-CKD exercise rehabilitation	HIIT versus MICT	Serum METRNL and cardiac-function measures	Intervention duration, attrition, and major exclusions NR	Between-group and longitudinal comparisons; covariate adjustment and multiplicity NR	Both groups improved; HIIT produced greater increases in LVEF and serum METRNL, whereas between-group changes in LVEDD and NT-proBNP were not significant. The human component does not establish skeletal-muscle source attribution or METRNL-mediated causality.
Panel B. Human observational or interventional studies in CKD or dialysis populations							
**Reference, study design, and evidence descriptor**	**Population and sample characteristics**	**Clinical or physiological context**	**Exposure/intervention and comparator**	**Key assessments**	**Follow-up, attrition, and exclusions**	**Statistical adjustment and multiplicity**	**Main finding and interpretive boundary**
Wang et al., 2025; multicenter cross-sectional study and longitudinal cohort; human observational evidence	Four Chinese centers; cross-sectional n=2,517, age 54.8 ± 14.0 years, 58.2% men; longitudinal n=544	HD or PD; longitudinal cohort enrolled at dialysis initiation	SMD/SMI categories and continuous measures versus TAC burden and progression	L1 CT-derived SMD/SMI; regional and total TAC	Cross-sectional N/A; longitudinal follow-up ≈3.5 years; exclusions NR	Multivariable regression and restricted cubic splines; adjusted demographic, cardiovascular, dialysis, inflammatory/lipid, and mineral-bone factors; baseline TAC included in progression models; multiplicity NR	Higher SMD/SMI were associated with lower TAC burden and slower progression. Observational imaging associations do not establish mediator transfer or molecular directionality.
Li et al., 2025; four-center retrospective cross-sectional study; human observational evidence	2,772 screened; 2,140 analyzed; age 55 ± 14 years; 58.8% men; 80.5% HD, 19.5% PD	Maintenance HD or PD; dialysis duration 3.5 ± 4.4 years	SMD/SMI quartiles and continuous measures versus no, single-valve, or dual-valve calcification	L1 CT-derived SMD/SMI; echocardiographic valve calcification and LVEF	Cross-sectional; dropout N/A; excluded acute HF/MI (73), malignancy (111), liver failure (50), parathyroidectomy (29), Alzheimer disease (2), and inadequate CT (367)	Ordinal logistic regression; adjusted demographic, dialysis, cardiometabolic, laboratory, mineral-bone, and medication factors; subgroup/sensitivity analyses and E-values; no multiplicity correction	Lower SMD, but not SMI, was associated with greater valve-calcification burden. Temporality and molecular transfer cannot be inferred; residual confounding and inter-scanner variability limit generalizability.
Li et al., 2026; exploratory human component of a mixed translational study; human observational evidence	CKD n=13; healthy controls n=6; detailed demographic and comorbidity data NR	CKD versus healthy controls; CKD-stage distribution NR	Cross-sectional comparison of circulating EVs and EV-associated small RNAs	EV characterization, small-RNA sequencing, and cardiac-injury markers	Cross-sectional; dropout N/A; exclusions NR	Exploratory omics analysis; limited confounder adjustment; omics multiplicity control reported	CKD-associated EV/miRNA profiles were identified, but the small exploratory sample cannot establish renal origin or cardiac causality. The findings suggest a possible kidney-to-heart pathway rather than skeletal muscle-to-heart signaling.
Wu et al., 2025; four-center cross-sectional study with indirect-effect analysis; human observational evidence	n=1,916; median age 56 years; 56.8% men	Maintenance HD or PD	SMD evaluated in relation to MLR, ALP, HDL/apolipoprotein A1, and TAC	Chest-CT SMD and TAC; inflammatory and metabolic indices	Cross-sectional; dropout N/A; exclusions NR	Multivariable and stratified regression; cross-sectional indirect-effect analysis; measured confounder adjustment; multiplicity NR	SMD statistically accounted for a small proportion of selected cross-sectional associations with TAC. The analysis does not establish temporal sequence, biological mediation, or molecular muscle-to-cardiovascular causality.
Sheng et al., 2023; four-center retrospective cohort; human observational evidence	642 screened; 485 included; median age 57 years; 62.5% men; 257 had both L1 and L3 images	Incident HD or PD	CT-derived SMD/SMI evaluated as mortality predictors	L1/L3 SMD and SMI; all-cause and cardiac mortality	Three years; excluded transplant (4), cancer (3), Alzheimer disease (1), inadequate CT (109); 40 lost to follow-up	Cox regression; adjusted demographic, BMI, smoking, diabetes, hypertension, coronary disease, laboratory, and medication factors; multiplicity NR	Lower L1 SMD, but not SMI, predicted all-cause and cardiac mortality. Retrospective selection, imaging exclusions, attrition, and unmeasured activity/diet limit inference; prognostic association does not demonstrate molecular crosstalk.
Zheng et al., 2025; multicenter retrospective cohort; human observational evidence	n=833; age 53.30 ± 13.35 years; 64.35% men; 615 HD, 218 PD	Incident HD or PD	Low-attenuation muscle phenotype evaluated in relation to mortality	L1 low-attenuation muscle indices and total muscle area; all-cause mortality	Median follow-up 3.86 years; 225 deaths; exclusions/attrition NR	Multivariable survival models; adjusted demographic, comorbidity, dialysis, and laboratory factors; multiplicity NR	Lower muscle adipose infiltration was associated with lower mortality. The finding is observational and metric-specific; no cardiac-specific endpoint or circulating mediator was assessed.
Graham-Brown et al., 2021, CYCLE-HD; open-label, endpoint-assessor-blinded cluster-randomized trial; human physiological or interventional evidence	n=130, 65 per group; age ≈57 years; ≈73% men	Maintenance HD	Six-month intradialytic cycling versus usual care	Cardiac MRI LV mass; secondary cardiac, vascular, arrhythmia, physical-function, and quality-of-life outcomes	Six months; 101/130 completed assessment; 29/130 did not complete	Cluster-randomized intention-to-treat analysis; missing-data sensitivity analyses; primary endpoint prespecified; secondary-outcome multiplicity NR	Cycling reduced LV mass. The trial supports an exercise-related cardiac imaging benefit, but did not evaluate myokines, EVs, or mediation of the cardiac response.
Ridker et al., 2021, RESCUE; multicenter double-blind placebo-controlled phase 2 trial; human physiological or interventional evidence	n=264; median age 68 years; 51% men; 71% diabetes; 48% established atherosclerosis	CKD stages 3–5; eGFR 10–59 mL/min/1.73 m²; hsCRP ≥2 mg/L	Ziltivekimab 7.5, 15, or 30 mg every 4 weeks versus placebo	hsCRP at 12 weeks; inflammatory/thrombotic biomarkers and safety; no muscle or cardiac-remodeling endpoint	Primary endpoint at 12 weeks; follow-up to 24 weeks; attrition NR	Prespecified randomized dose-group comparisons with multiplicity adjustment	Ziltivekimab reduced inflammatory biomarkers. This provides indirect translational support for IL-6 inhibition, but not evidence for muscle-derived IL-6, muscle–cardiovascular communication, cardiac remodeling, or cardiovascular events.

Human studies directly underpinning the principal physiological, exercise-related, CKD-associated, or translational claims of this review are summarized. For mixed translational studies, only the human component is presented in **Table 2**; the corresponding cellular or animal components are discussed in the relevant mechanistic sections. NR, not reported in the primary publication; N/A, not applicable.

In this review, we use “hierarchical signaling networks” to describe the organization of CKD-related skeletal muscle–heart communication across three interconnected levels. The upstream level comprises CKD-related systemic stressors, including uremic toxin retention, chronic inflammation, oxidative stress, hypoxia, metabolic disturbance, and neurohumoral activation. The intermediate level includes circulating mediators such as myokines, cytokines, EVs, and EV-associated miRNA/protein cargoes. The downstream level refers to cardiac target-cell responses, including cardiomyocyte metabolic reprogramming, fibroblast activation, endothelial dysfunction, extracellular matrix deposition, and progressive cardiac remodeling. These levels are not strictly linear, because cardiac-derived endocrine factors, impaired peripheral perfusion, and neurohumoral activation may feed back on skeletal muscle and further aggravate catabolism.

## 2 Physiological crosstalk between skeletal muscle and the heart

Under physiological conditions, skeletal muscle and the heart communicate to maintain metabolic and circulatory homeostasis. At rest, skeletal muscle releases myokines, metabolic mediators, and EVs at relatively low and dynamically regulated levels, contributing to the regulation of myocardial metabolism and vascular tone. In concert, the heart meets the basal metabolic demands of skeletal muscle by adjusting perfusion, heart rate, and stroke volume, thereby maintaining circulatory homeostasis (Walzik et al., 2024; Iglesias, 2025; Shero et al., 2025).

Skeletal muscle is increasingly recognized as an endocrine organ that releases diverse myokines with systemic actions on cardiovascular tissues. A representative example is myostatin, a member of the transforming growth factor-β (TGF-β) superfamily, which is produced predominantly by skeletal muscle and, to a lesser extent, by the myocardium under pathological stress. Myostatin primarily signals through binding to activin receptor type IIB (ActRIIB) and activating the Smad2/3 pathway, thereby limiting myofiber hypertrophy. Counterbalancing this axis, follistatin (FST) binds and neutralizes myostatin/activin signaling, helping to preserve muscle mass and support muscle growth. The balance between myostatin and FST contributes to the maintenance of muscle mass and energy homeostasis (Chow et al., 2022; Walzik et al., 2024; Shero et al., 2025). Angiopoietin-like protein 4 (ANGPTL4) can regulate systemic fatty acid supply and partitioning by inhibiting lipoprotein lipase activity, thereby providing the myocardium with a more stable substrate milieu for energy utilization. In addition, during habitual daily activity, skeletal muscle releases mediators such as IL-15 and IL-7, which contribute to the maintenance of metabolic and immune homeostasis. Beyond myokines, both skeletal muscle and the myocardium release extracellular vesicles (EVs). These vesicles carry proteins, microRNAs (miRNAs), and other bioactive cargoes and can mediate paracrine and endocrine signaling (Kumar et al., 2024). Experimental studies suggest that skeletal muscle-derived EVs can influence cardiovascular tissues; however, direct *in vivo* evidence that physiologically released muscle EVs reach cardiomyocytes and alter their function remains limited (Chen et al., 2025).

The heart also communicates with skeletal muscle through endocrine signals. Cardiac natriuretic peptides provide a direct physiological example. Human skeletal muscle expresses natriuretic peptide receptor A (NPRA), also known as guanylyl cyclase-A (GC-A), and atrial natriuretic peptide (ANP) and B-type natriuretic peptide (BNP) activate cGMP-dependent signaling in primary human myotubes, increasing PGC-1α expression, oxidative phosphorylation gene and protein expression, fatty acid oxidation, and maximal respiration (Engeli et al., 2012). Subsequent genetic evidence showed that reduced ANP–GC-A signaling impairs skeletal muscle mitochondrial function and endurance capacity *in vivo (*Carper et al., 2024). Thus, cardiac natriuretic peptides constitute an endocrine component of physiological heart-to-muscle communication. The supporting evidence includes direct ANP/BNP exposure in differentiated primary human myotubes and an 8-week human exercise-training component, together with systemic loss-of-function models and AAV9-mediated, skeletal muscle-targeted partial GCA knockdown in mice. These studies demonstrate recipient-muscle responsiveness and ANP–GC-A pathway involvement but do not independently establish a complete *in vivo* human heart-to-muscle signaling axis or CKD-specific relevance. The human exercise-training component is summarized in **Table 2**.

**Figure 1:**
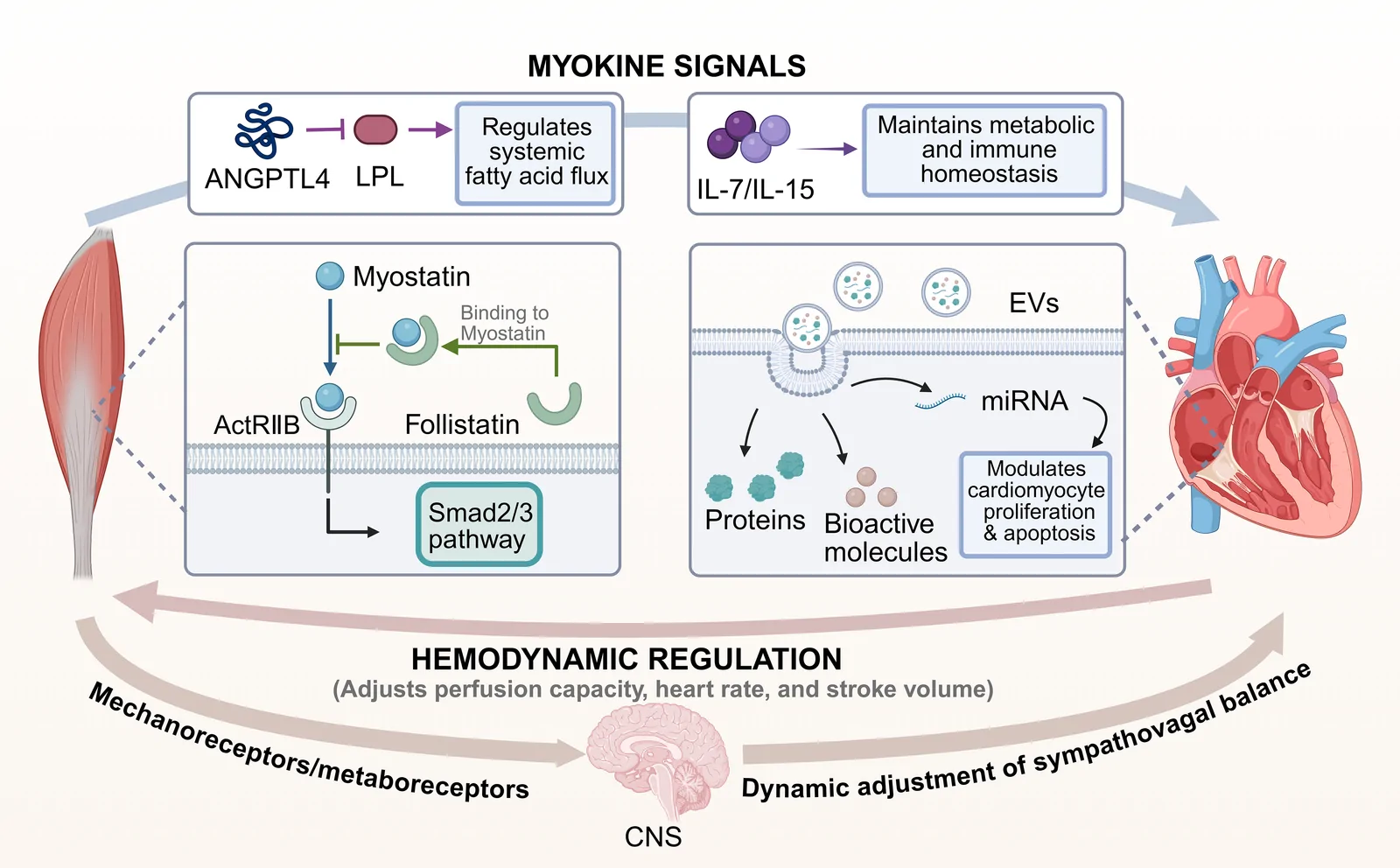
Physiological skeletal muscle–heart crosstalk. Skeletal muscle communicates with the heart through myokines, extracellular vesicles (EVs), and hemodynamic-neural regulation. Myokines such as ANGPTL4, IL-7/IL-15, and the myostatin-follistatin axis regulate fatty acid flux, metabolic and immune homeostasis, and Smad2/3 signaling. Skeletal muscle-derived EVs carry miRNAs, proteins, and other bioactive molecules that may modulate cardiomyocyte proliferation and apoptosis. In parallel, mechano- and metaboreceptor feedback to the central nervous system helps adjust perfusion, heart rate, stroke volume, and sympathovagal balance, thereby maintaining physiological muscle-heart homeostasis. Conversely, cardiac-derived natriuretic peptides may act on skeletal muscle through NPRA/GC-A–cGMP signaling to support oxidative metabolism and mitochondrial function.

Alongside this endocrine route, the heart influences skeletal muscle through rapid hemodynamic and neurohumoral regulation. By rapidly adjusting perfusion, heart rate, and stroke volume, the heart matches blood flow and oxygen delivery to the energetic needs of skeletal muscle. In parallel, skeletal muscle senses local changes in its mechanical and metabolic milieu via mechano- and metaboreceptors and relays these inputs to central cardiovascular control networks. This, in turn, modulates sympathetic-vagal balance and dynamically shapes heart rate, myocardial contractility, and peripheral vascular resistance, enabling rapid closed-loop regulation (Mitchell and Victor, 1996; Iannetta et al., 2025; O'Leary and Mannozzi, 2025). These physiological endocrine, vesicular, neural, and hemodynamic pathways are summarized in **Figure 1**.

Under physiological conditions, skeletal muscle–heart signaling is generally low-amplitude, short-lived, and reversible, supporting metabolic and circulatory homeostasis. Against this baseline, the transition to exercise amplifies skeletal muscle-derived signals, including myokines, within a short time frame and, together with neurohumoral control, drives coordinated adjustments in cardiac perfusion, thereby establishing more robust inter-organ communication. Accordingly, the next section focuses on the mechanisms and key mediators that strengthen skeletal muscle–heart interactions during exercise.

## 3 Exercise-induced interactions between skeletal muscle and the heart

**Figure 2:**
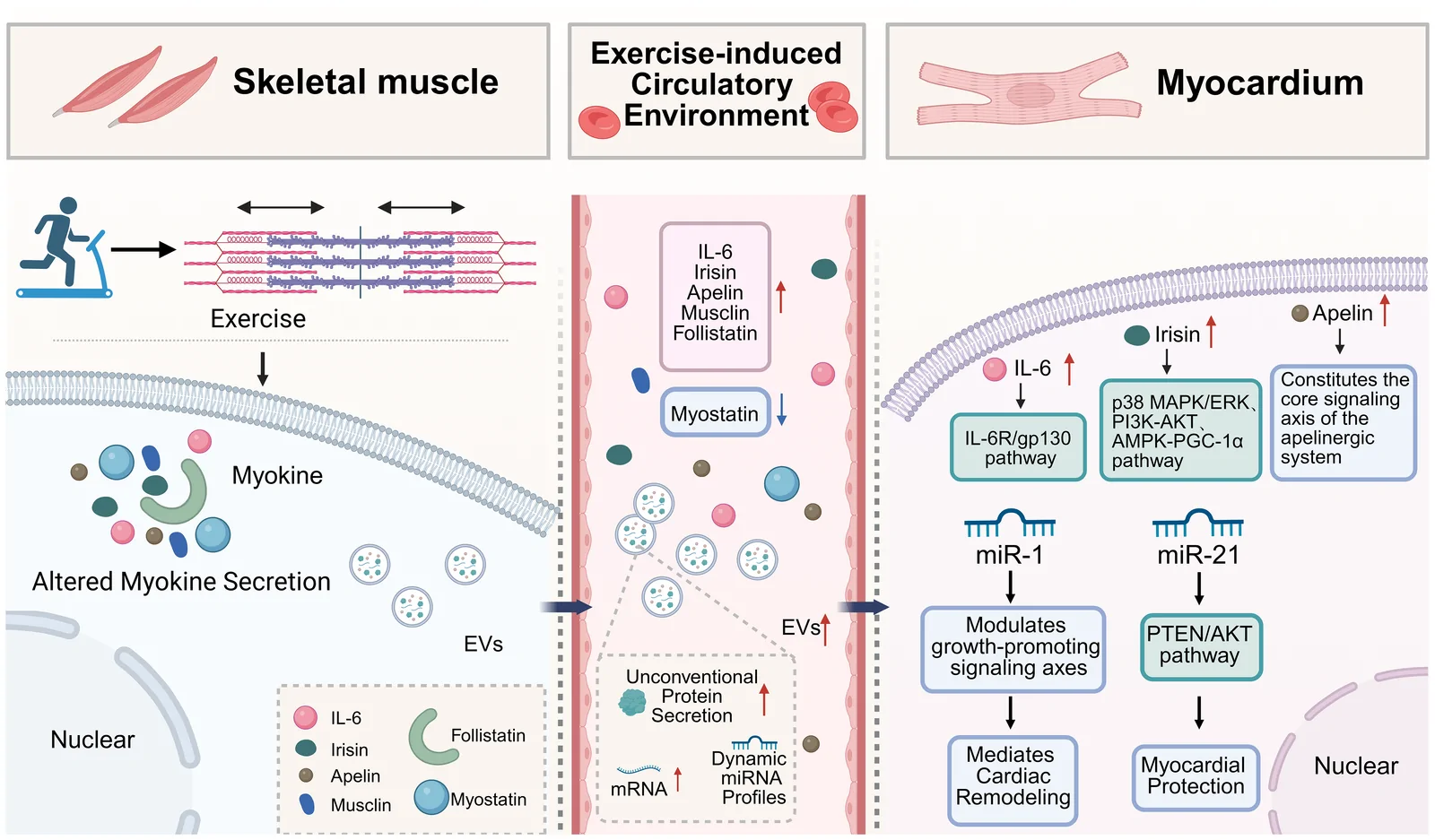
Exercise-induced skeletal muscle–heart crosstalk. Exercise enhances skeletal muscle-derived myokine secretion and EV release, creating a transient circulatory environment that supports communication with the myocardium. Increased IL-6, irisin, apelin, musclin, and follistatin, reduced myostatin signaling, and EV-associated miRNA/mRNA/protein cargoes may regulate myocardial metabolic adaptation, stress responses, cardioprotection, and remodeling through pathways including IL-6R/gp130, p38 MAPK/ERK, PI3K-AKT, AMPK-PGC-1α, apelinergic signaling, and miR-1/miR-21-related axes.

Exercise transiently amplifies physiological skeletal muscle–heart communication. Skeletal muscle contraction increases the release of myokines and can also alter the abundance and molecular cargoes of circulating extracellular vesicles (Chow et al., 2022; Robbins and Gerszten, 2023; Shero et al., 2025). In parallel, exercise induces rapid hemodynamic and autonomic adjustments, including central command, mechano- and metaboreflex activation, and sympathovagal modulation, which together promote perfusion redistribution, substrate mobilization, and cardiovascular workload matching (Nobrega et al., 2014; Wan et al., 2023). Depending on exercise intensity and duration, circulating neurohumoral mediators, including catecholamines and renin–angiotensin–aldosterone system-related signals, may further contribute to vascular tone, fluid balance, and substrate availability (Robbins and Gerszten, 2023; Shero et al., 2025). These responses enable the myocardium to meet increased energetic demand while preserving metabolic flexibility and mitochondrial function. After exercise cessation, most signals gradually return toward baseline; however, repeated stimulation through regular training can reduce systemic inflammatory burden, improve metabolic homeostasis, and enhance stress tolerance (Chow et al., 2022; Robbins and Gerszten, 2023). These adaptive effects are closely linked to coordinated changes in myokine signaling, EV/miRNA-related pathways, and neurohumoral regulation (**Figure 2**).

### 3.1 Myokines

Myokines are major mediators released by contracting skeletal muscle during exercise. Skeletal muscle contraction can rapidly increase circulating myokine concentrations. Together with neurohumoral adjustments, this response plays an important role in shaping exercise-related skeletal muscle–heart crosstalk. Notably, the magnitude and duration of myokine responses vary substantially across exercise modalities and training intensities. The major exercise-associated muscle-derived mediators discussed in this section are summarized in **Table 1**, whereas relevant human physiological, exercise, and interventional studies are summarized in **Table 2**.

#### 3.1.1 Interleukin-6

IL-6 is one of the earliest identified and most extensively studied exercise-responsive myokines. During exercise, skeletal muscle contraction can induce a transient increase in circulating IL-6, with the magnitude and duration of this response depending on exercise intensity, duration, recruited muscle mass, and energy availability (Kistner et al., 2022; Nash et al., 2023). This temporal pattern is relevant to skeletal muscle–heart communication because exercise-induced IL-6 is short-lived and metabolically coupled, which distinguishes it from sustained inflammatory IL-6 signaling (Kistner et al., 2022).

At the receptor level, IL-6 acts mainly through IL-6R/gp130-dependent signaling, activating STAT3 and MAPK-related pathways in metabolic and cardiovascular tissues (McGinnis et al., 2015; Osman and Neamati, 2024). IL-6 can also activate AMPK in skeletal muscle and adipose tissue, linking this cytokine to glucose uptake, fatty acid mobilization, and substrate redistribution during exercise (Kelly et al., 2009). In the heart, gp130–STAT3-related signaling has been associated with cardiomyocyte survival, mitochondrial adaptation, and protection against excessive stress responses (McGinnis et al., 2015; Osman and Neamati, 2024). Exercise-induced IL-6 is also frequently accompanied by anti-inflammatory effects, including induction of IL-10 and soluble TNF receptors and suppression of TNF-α-related inflammatory activity (Baskerville et al., 2024). Therefore, IL-6 is best interpreted as a context-dependent mediator within exercise-induced skeletal muscle–heart crosstalk: transient, contraction-coupled IL-6 signaling may contribute to cardiometabolic adaptation, whereas sustained inflammatory IL-6 signaling is more closely associated with endothelial dysfunction, fibro-inflammatory activation, and adverse remodeling (Kistner et al., 2022; Osman and Neamati, 2024). Mechanistic support for exercise-related IL-6 cardioprotection is derived primarily from an acute murine exercise-preconditioning model followed by myocardial ischemia–reperfusion. This design supports an IL-6-dependent component of acute exercise-induced cardioprotection but does not establish skeletal muscle as the exclusive IL-6 source and should not be directly extrapolated to chronic CKD-associated metabolic dysfunction or fibrotic remodeling.

#### 3.1.2 Irisin/FNDC5

Irisin, a cleaved product of fibronectin type III domain-containing protein 5 (FNDC5), is induced by exercise-associated activation of the PGC-1α–FNDC5 axis in skeletal muscle (Boström et al., 2012). Since its initial description as an exercise-responsive myokine, irisin has been widely studied in relation to mitochondrial homeostasis, metabolic remodeling, thermogenic energy expenditure, and cardiovascular protection (Maak et al., 2021). In cardiovascular and metabolic tissues, irisin has been reported to activate AMPK, Akt, ERK, p38 MAPK, and PGC-1α-related pathways (Zhao, 2022). αV-class integrin-dependent signaling has been proposed as one potential receptor mechanism for irisin (Kim et al., 2018).

Through these pathways, irisin may enhance mitochondrial biogenesis, improve glucose and lipid handling, reduce oxidative stress, and support cardiomyocyte survival under metabolic stress (Chen et al., 2019; Li et al., 2024). In experimental models of myocardial injury and remodeling, irisin has also been associated with attenuation of inflammatory activation, suppression of TGF-β1–Smad2/3-related fibrotic signaling, and reduced extracellular matrix deposition (Chen et al., 2019; Li et al., 2024). These observations provide a plausible mechanistic basis by which exercise-induced skeletal muscle signaling may influence myocardial metabolic adaptation and anti-fibrotic remodeling. However, circulating irisin responses to aerobic exercise, resistance training, and high-intensity interval training (HIIT) remain heterogeneous, partly because of differences in exercise prescriptions, sampling time, population characteristics, and assay methodology (Zhao, 2022). Thus, irisin remains a promising but methodologically complex mediator of exercise-induced skeletal muscle–heart communication. The principal antifibrotic evidence discussed here was obtained in a non-CKD myocardial infarction model using global *Fndc5*-deficient mice undergoing resistance exercise, together with experiments in primary cardiac fibroblasts (Li et al., 2024). These findings support the involvement of the FNDC5/irisin pathway but do not establish skeletal muscle-derived circulating irisin as the sole mediator of the cardiac response.

#### 3.1.3 Apelin

Apelin is the endogenous ligand of the APJ receptor and is expressed in skeletal muscle as well as cardiovascular and metabolic tissues (Tatemoto et al., 1998; Rozwadowski et al., 2022). Accumulating evidence suggests that exercise can upregulate apelin expression in skeletal muscle, supporting its potential role in skeletal muscle–circulation–target organ communication (Kilpiö et al., 2024). In metabolic tissues, the apelin–APJ pathway promotes glucose uptake, fatty acid oxidation, mitochondrial metabolism, and insulin sensitivity through AMPK-, PI3K–Akt-, and related signaling pathways (Kilpiö et al., 2024; Walzik et al., 2024).

Within the cardiovascular system, apelin has been linked to endothelium-dependent vasodilation, nitric oxide bioavailability, angiogenesis, positive inotropy, improved myocardial perfusion, and contractile adaptation (Chapman et al., 2023; Tang et al., 2024). These vascular and metabolic actions are directly relevant to exercise-induced cardiac adaptation because they may improve substrate delivery, oxygen utilization, and workload matching. In experimental cardiovascular disease models, apelin has also been linked to attenuation of oxidative stress, inflammatory activation, and profibrotic signaling, including TGF-β/Smad-related pathways (Lv et al., 2020). Therefore, apelin may serve as a bridge between exercise-induced skeletal muscle signaling, vascular adaptation, myocardial energy metabolism, and structural remodeling. However, the exercise-related regulation of skeletal muscle apelin and the cardiac effects of apelin have largely been demonstrated in separate experimental settings. Key cardiovascular studies used exogenous apelin delivery or non-CKD disease models, demonstrating cardiac responsiveness to apelin rather than direct endogenous skeletal muscle-to-heart apelin transfer.

#### 3.1.4 Musclin (osteocrin)

Musclin, also known as osteocrin, is a secreted peptide highly expressed in skeletal muscle and regulated by exercise and energy status (Chow et al., 2022; Szaroszyk et al., 2022). Mechanistically, musclin can bind the natriuretic peptide clearance receptor NPR-C, thereby reducing natriuretic peptide clearance and enhancing ANP/BNP/CNP signaling (Kanai et al., 2017; Szaroszyk et al., 2022). This axis is relevant to skeletal muscle–heart communication because natriuretic peptide signaling can modulate cardiomyocyte contractility and suppress cardiac fibroblast activation mainly through cyclic nucleotide-dependent pathways, particularly cGMP/PKG signaling (Szaroszyk et al., 2022; Sarzani et al., 2024). Experimental evidence suggests that reduced skeletal muscle musclin expression may worsen cardiac dysfunction under stress, whereas musclin supplementation or upregulation may attenuate myocardial fibrosis and improve cardiac performance (Szaroszyk et al., 2022; Saw et al., 2025). Therefore, musclin may connect exercise-conditioned skeletal muscle with cardiac natriuretic peptide responsiveness, although its role in human exercise-induced cardiac adaptation remains less established than that of IL-6 or irisin (Saw et al., 2025). Musclin is supported by comparatively direct source-specific evidence: skeletal muscle-specific *Ostn* loss aggravated, whereas muscle-directed *Ostn* overexpression attenuated, cardiac dysfunction and fibrosis during pressure overload. Nevertheless, these findings were obtained in a non-CKD model, and the overexpression experiment represents therapeutic gain of function rather than physiological human exposure.

#### 3.1.5 Myostatin–Follistatin

The myostatin–follistatin axis primarily reflects the balance between inhibitory and permissive signals for skeletal muscle remodeling. Regular resistance training is generally associated with reduced myostatin signaling and increased follistatin activity, paralleling improvements in muscle mass and strength (Rodgers and Ward, 2022). By shifting this balance, exercise creates a systemic environment more favorable for muscle anabolism, metabolic flexibility, and exercise capacity (Rodgers and Ward, 2022; Khalafi et al., 2023). These changes may indirectly benefit the heart by improving peripheral substrate handling, reducing metabolic stress, and enhancing cardiorespiratory reserve (Robbins and Gerszten, 2023; Hastings et al., 2024). However, compared with its well-established role in skeletal muscle adaptation, the direct contribution of exercise-regulated myostatin–follistatin signaling to myocardial remodeling remains less clearly defined (Shi et al., 2023; Hastings et al., 2024).

#### 3.1.6 Other myokines

Other exercise-responsive myokines may also contribute to skeletal muscle–heart communication. Follistatin-like protein 1 (FSTL1) can be induced by resistance exercise and has been reported to exert anti-apoptotic effects on cardiomyocytes through PI3K–Akt–mTOR signaling and modulation of the Bcl-2/Bax axis (Oshima et al., 2008; Xi et al., 2021). Experimental studies also suggest that FSTL1 may reduce myocardial fibrosis and collagen deposition and improve myocardial metabolism (Seki et al., 2018). Meteorin-like protein (METRNL) is increased by HIIT in skeletal muscle and may act on the myocardium through circulating routes (Reboll et al., 2022; Wang et al., 2024). Mechanistically, METRNL has been linked to cardiomyocyte AMPK activation, HDAC4 phosphorylation, GLUT4 expression, glucose utilization, and mitochondrial respiratory function (Reboll et al., 2022; Wang et al., 2024). The supporting METRNL study combined an exercise intervention in patients with heart failure with transverse-aortic-constriction models, *Metrnl* manipulation, exogenous METRNL treatment, and cardiomyocyte experiments. The human component is summarized in **Table 2**. Although the combined findings support METRNL pathway involvement, they do not independently trace endogenous skeletal muscle-derived METRNL to cardiac target cells, and the experimental models were not CKD-specific. Additional mediators, including BDNF and IGF-1, may further modulate substrate metabolism, stress tolerance, and tissue repair (Ji et al., 2024; Lee et al., 2024). These mediators indicate that exercise-induced myokine signaling is organized as a network rather than dominated by a single ligand (Chow et al., 2022; Iannetta et al., 2025).

### 3.2 EVs and miRNAs

Beyond myokines, exercise-induced EVs and their molecular payloads, particularly miRNAs, represent an additional and increasingly recognized route for exercise-associated inter-organ signaling relevant to skeletal muscle–heart communication (Whitham et al., 2018; Denham and Spencer, 2020). EVs are membrane-bound particles that encapsulate miRNAs, proteins, lipids, and other nucleic acids and remain relatively stable in biofluids (Théry et al., 2018; Kalluri and LeBleu, 2020). During exercise, EVs may be released by multiple cell types, including skeletal muscle cells, endothelial cells, immune cells, and cardiomyocytes, thereby complicating source attribution (van Niel et al., 2018; Welsh et al., 2024).

Secretomic studies of cultured human myotubes support the concept that skeletal muscle can release proteins, RNAs, and vesicular cargoes with potential inter-organ signaling capacity (Hoffmann and Weigert, 2017; Förster et al., 2025). Consistently, exercise-associated circulating EVs have been reported to carry non-classically secreted proteins and mRNAs, suggesting that EVs may serve as carriers for long-distance signal transfer during exercise (Whitham et al., 2018). During exercise, circulating EV abundance can rise rapidly, and release kinetics are closely coupled to exercise intensity and metabolic load (Frühbeis et al., 2015). Meanwhile, circulating miRNAs exhibit dynamic changes across endurance exercise, resistance training, and HIIT, with these shifts linked to energy metabolism, inflammatory regulation, and tissue adaptation (Sanchis-Gomar et al., 2021). Functionally, several studies have associated exercise-induced circulating EVs with cardioprotection; for example, in ischemia–reperfusion settings, they may attenuate myocardial injury and facilitate recovery (Bei et al., 2017; Hou et al., 2019). Running interventions can also reshape circulating miRNA profiles, and differentially expressed miRNAs have been suggested to regulate MAPK-related pathways (Oliveira et al., 2018). Given the bidirectional roles of MAPK signaling in cardiac development, functional maintenance, and disease progression, these observations indicate that exercise-related remodeling of EV/miRNA signatures may bridge acute exercise stimuli and longer-term myocardial phenotypic adaptation (Zhang et al., 2024; Wang X. et al., 2025).

At the level of individual miRNAs, striated muscle-enriched miRNAs such as miR-1 and miR-133a/b have been repeatedly implicated in exercise adaptation and cardiovascular phenotypes. In the context of myocardial hypertrophy, miR-1 expression is frequently downregulated and has been linked to IGF-1-related growth and metabolic signaling, including the IGF-1R–PI3K–Akt–mTOR axis (Sanchis-Gomar et al., 2021). miR-21 has been reported to participate in ischemia–reperfusion responses via the PTEN/Akt pathway and may exert post-injury cardioprotective effects, at least in part, through modulation of apoptosis-related processes (Huang et al., 2017; Surina et al., 2021). Exercise-associated EV and miRNA changes may complement soluble myokines and prolong inter-organ signaling beyond the exercise bout. Nevertheless, because circulating EVs arise from multiple tissues during exercise, future studies require better source-tracing strategies to define which EV populations are truly skeletal muscle-derived and which cardiac effects are directly mediated by their cargoes (Whitham et al., 2018; Welsh et al., 2024). Human exercise studies demonstrate acute changes in circulating EV abundance and EV-associated protein cargoes, as summarized in **Table 2**. However, these studies used bulk circulating EV preparations and did not establish that the measured EVs originated predominantly from skeletal muscle or were delivered to cardiac target cells. Experimental transfer studies support the cardioprotective potential of exercise-associated circulating EVs, but these EVs represent a multisource circulating pool rather than source-specific skeletal muscle-to-heart evidence.

### 3.3 Hemodynamic and neurohumoral coupling during exercise

Exercise-induced skeletal muscle–heart communication is not mediated solely by secreted molecules. Rapid hemodynamic and neurohumoral regulation provides an essential framework within which myokines and EV-associated signals act (Nobrega et al., 2014; Wan et al., 2023). During exercise, skeletal muscle contraction increases local metabolic demand and activates mechano- and metaboreceptor afferents, which relay information to central cardiovascular control networks. This exercise pressor reflex, together with central command, increases sympathetic outflow and withdraws parasympathetic tone, thereby raising heart rate, myocardial contractility, venous return, and peripheral vascular redistribution. These responses allow cardiac output to match skeletal muscle oxygen and substrate demand while maintaining arterial pressure.

At the same time, increased shear stress and endothelial activation promote vasodilatory responses in active skeletal muscle, whereas neurohumoral signals help mobilize glucose and fatty acids from liver and adipose tissue (Robbins and Gerszten, 2023; Shero et al., 2025). Depending on exercise duration and intensity, catecholamines and RAAS-related mediators may further regulate vascular tone, fluid balance, and substrate availability (Chow et al., 2022; Shero et al., 2025). Therefore, neurohumoral regulation should be viewed as a bidirectional interface between skeletal muscle and the heart: skeletal muscle-derived afferent signals shape cardiovascular output, while cardiac output and vascular regulation determine the perfusion and metabolic environment of contracting muscle (Nobrega et al., 2014; Wan et al., 2023). This dynamic loop helps explain how exercise converts local skeletal muscle contraction into systemic cardiovascular adaptation.

Taken together, exercise-induced skeletal muscle–heart communication is characterized by transient, contraction-coupled, and reversible signaling. Myokines, EV-associated cargoes, neurohumoral activation, and hemodynamic adjustments are generated within a defined physiological window and generally subside during recovery (Chow et al., 2022; Shero et al., 2025). This physiological pattern provides a useful reference for interpreting CKD-associated changes. As discussed in the following section, CKD may reshape these same communication routes into more persistent, non-contraction-dependent, and cellularly heterogeneous signals, thereby shifting skeletal muscle–heart interactions from adaptive coordination toward maladaptive remodeling.

## 4 CKD-driven maladaptive skeletal muscle–heart crosstalk

**Figure 3:**
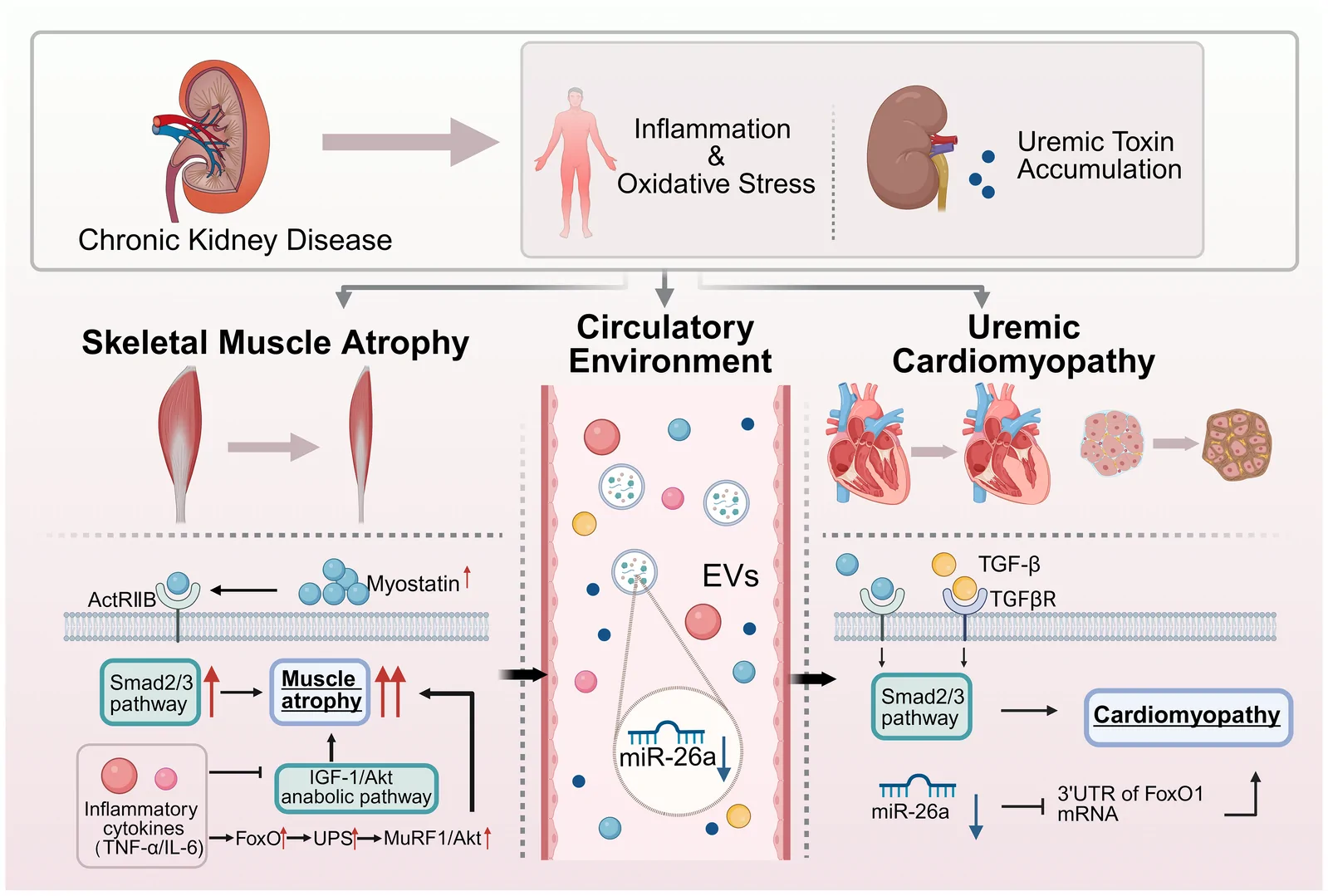
CKD-driven maladaptive skeletal muscle–heart crosstalk. Chronic kidney disease promotes a uremic circulatory environment marked by toxin accumulation, inflammation, and oxidative stress. These stressors drive skeletal muscle atrophy through myostatin-ActRIIB-Smad2/3 signaling, inflammatory cytokines, impaired IGF-1/Akt anabolic signaling, and FoxO-related proteolytic pathways. Altered EV and miRNA cargoes may transmit maladaptive signals to the myocardium, whereas restoration of protective miR-26a signaling has been shown to attenuate skeletal muscle wasting and cardiac fibrosis in CKD models.

Unlike exercise-induced signaling, which is transient, contraction-coupled, and reversible, CKD imposes persistent, non-contraction-dependent stress on both skeletal muscle and the heart, converting physiological communication into maladaptive crosstalk. At the upstream level, uremic toxin retention, chronic inflammation, oxidative stress, hypoxia, metabolic acidosis, insulin resistance, hemodynamic overload, and neurohumoral activation affect both skeletal muscle and the myocardium (Patel et al., 2022; Edwards et al., 2026). At the intermediate level, these systemic disturbances alter myokine secretion, inflammatory signaling, EV release, and miRNA cargo composition (Grange and Bussolati, 2022; Ng et al., 2025). At the downstream level, cardiomyocytes, cardiac fibroblasts, and endothelial cells translate these inputs into metabolic inflexibility, mitochondrial dysfunction, microvascular impairment, extracellular matrix deposition, and progressive cardiac dysfunction. This framework provides a mechanistic basis for linking CKD-associated sarcopenia with uremic cardiomyopathy and fibrotic remodeling (**Figure 3**).

### 4.1 Upstream CKD-related systemic drivers

CKD establishes a persistent uremic, inflammatory, and metabolic environment that affects skeletal muscle, the myocardium, and the circulation. Cardiovascular manifestations of CKD include left ventricular hypertrophy, diastolic dysfunction, microvascular abnormalities, and myocardial fibrosis (House et al., 2019; Wang and Shapiro, 2019). These phenotypes reflect overlapping exposure to uremic toxin retention, impaired oxygen delivery, oxidative stress, insulin resistance, metabolic acidosis, hemodynamic overload, and neurohumoral activation (Patel et al., 2022; Curaj et al., 2024). Interstitial fibrosis and microvascular dysfunction may emerge before advanced uremia and become progressively compounded by hypertrophy, impaired perfusion, and declining kidney function (Edwards et al., 2026).

These disturbances act upstream by altering both the signal-producing skeletal muscle and the signal-receiving myocardium. In skeletal muscle, inflammatory and metabolic stress impairs mitochondrial function, suppresses IGF-1–Akt-dependent protein synthesis, activates FoxO-regulated proteolysis, and limits regenerative capacity (Cohen et al., 2015; Sabatino et al., 2021). These changes promote muscle wasting and may also modify the skeletal muscle secretory phenotype. In the heart, the same systemic environment reduces metabolic flexibility, impairs mitochondrial oxidative capacity, promotes endothelial dysfunction, and activates profibrotic signaling (Patel et al., 2022; Edwards et al., 2026). Thus, CKD-related crosstalk is more likely to result from converging systemic stressors than from one dominant circulating mediator.

### 4.2 Dysregulated myokine, cytokine, and anabolic–catabolic signaling

At the intermediate level, CKD may reprogram skeletal muscle-derived and systemic myokine-related signaling. The myostatin/activin–ActRIIB–Smad2/3 axis is one of the most relevant pathways linking muscle catabolism with potential cardiac remodeling. In skeletal muscle, activation of this pathway suppresses anabolic signaling, promotes proteolysis, and contributes to CKD-associated muscle wasting (Verzola et al., 2019). At the cardiac level, Smad2/3-dependent signaling overlaps with canonical TGF-β-driven profibrotic pathways, making this axis a plausible point of convergence between muscle catabolism and myocardial fibrotic remodeling (Travers et al., 2016; Castillero et al., 2020). However, circulating myostatin or activin levels alone do not establish skeletal muscle as the dominant tissue source, because these mediators may also originate from the heart, kidney, inflammatory cells, or other tissues. Importantly, myostatin inhibition improved skeletal muscle mass and function in a pressure-overload model but did not attenuate cardiac hypertrophy or fibrosis. This finding indicates that improvement in skeletal muscle phenotype does not necessarily predict parallel improvement in myocardial remodeling.

Chronic inflammation further strengthens the link between muscle catabolism and cardiac injury. Persistent elevation of IL-6, TNF-α, and related cytokines in CKD differs fundamentally from the transient, contraction-coupled myokine response observed during exercise (Kistner et al., 2022). Chronic inflammatory signaling can impair insulin sensitivity, suppress mitochondrial oxidative metabolism, activate proteolytic pathways, and promote endothelial and fibro-inflammatory activation. In skeletal muscle, these effects reinforce catabolism and reduce regenerative capacity (Cohen et al., 2015). In the heart, persistent inflammatory signaling may interact with oxidative stress and neurohumoral activation to aggravate mitochondrial dysfunction, endothelial injury, and extracellular matrix remodeling (Travers et al., 2016; Patel et al., 2022). Thus, the same mediator may exert distinct biological effects depending on exposure duration, tissue source, receptor context, and the inflammatory milieu.

The IGF-1–Akt–FoxO axis provides another link between skeletal muscle wasting and cardiac vulnerability. In CKD-associated sarcopenia, impaired IGF-1–Akt signaling reduces protein synthesis and permits FoxO-dependent transcription of atrophy-related genes (Cohen et al., 2015). Similar Akt–FoxO and GSK-3β-related signaling abnormalities have been implicated in cardiac insulin resistance, mitochondrial dysfunction, and maladaptive remodeling (Patel et al., 2022; Curaj et al., 2024). These shared pathways suggest that CKD-associated muscle and cardiac abnormalities may reflect both inter-organ signaling and parallel responses to systemic stress. Therefore, dysregulated myokine signaling should be interpreted within a broader network that includes inflammatory, metabolic, and neurohumoral drivers rather than as an isolated skeletal muscle-to-heart pathway.

### 4.3 EV- and miRNA-mediated maladaptive inter-organ signaling

EVs and miRNA cargoes represent another intermediate layer of CKD-associated skeletal muscle–heart communication. CKD can alter the circulating EV pool and reshape EV-associated miRNA signatures, with potential links to inflammation, fibrosis, endothelial dysfunction, and cardiovascular injury (Grange and Bussolati, 2022; Ng et al., 2025). Specific EV cargoes have further been linked to vascular calcification and cardiac injury (Koide et al., 2023; Li et al., 2026). Because EVs can protect RNA cargoes from degradation and deliver regulatory molecules to recipient cells, they provide a plausible mechanism for sustained inter-organ signal transfer. However, plasma EVs are highly heterogeneous and may originate from the kidney, immune cells, platelets, endothelium, skeletal muscle, myocardium, and other tissues (Grange and Bussolati, 2022). Altered circulating EV cargo alone does not prove skeletal muscle-to-heart transfer; source labeling or molecular attribution is required.

The clearest intervention-based evidence for a manipulable muscle–heart EV-related pathway in CKD comes from the miR-26a study. In CKD models, reduced miR-26a expression has been observed in skeletal muscle and the heart (Wang B. et al., 2019). Intramuscular administration of engineered muscle satellite cell-derived exosomes enriched in miR-26a increased cardiac and skeletal muscle miR-26a levels, inhibited FoxO1-dependent insulin resistance, reduced muscle atrophy and cardiac fibrosis, and improved cardiac function (Wang B. et al., 2019). This study supports the therapeutic potential of EV-mediated inter-organ transfer. However, it should be distinguished from evidence that endogenous skeletal muscle-derived EVs are a dominant pathological signal in CKD. In this setting, loss of protective miR-26a and its experimental restoration, rather than excess pathogenic miR-26a, appears to be the relevant mechanism. Because the study used engineered muscle satellite cell-derived exosomes enriched in miR-26a, it represents exogenous or engineered-delivery evidence. It demonstrates that therapeutic restoration can improve skeletal muscle and cardiac phenotypes but does not establish endogenous skeletal muscle-derived EVs as the dominant physiological or pathological carrier in CKD.

Parallel EV pathways further illustrate why tissue-of-origin attribution is critical. Macrophage-derived exosomal miR-155 can induce cardiomyocyte pyroptosis and hypertrophic remodeling in uremic cardiomyopathy (Wang et al., 2020b). More recently, circulating EVs from patients and mice with CKD were shown to contain kidney-derived miRNAs that induced cardiomyocyte apoptosis, impaired contractile gene expression and calcium-handling pathways, and correlated with cardiac injury markers (Li et al., 2026). These studies support experimentally tractable macrophage-to-heart and kidney-to-heart EV pathways in the studied models, but they should not be interpreted as evidence of skeletal muscle-derived EV transfer. Instead, they indicate that multiple non-muscle EV pathways may converge on the myocardium and modify, mimic, or confound the skeletal muscle–heart axis.

### 4.4 Cardiac target-cell responses

At the downstream level, CKD-altered circulating mediators are translated into cell-specific cardiac responses. In cardiomyocytes, uremic toxins, inflammatory mediators, neurohumoral activation, and EV-associated cargoes may impair insulin signaling, reduce Akt activity, alter GSK-3β and FoxO1 signaling, and limit the ability to switch between fatty acid and glucose utilization (Patel et al., 2022; Curaj et al., 2024). These abnormalities reduce metabolic flexibility and mitochondrial oxidative capacity, increase reactive oxygen species generation, disturb calcium handling, and lower tolerance to hemodynamic or ischemic stress. EV-associated kidney-derived miRNAs have also been linked to impaired contractile gene expression and calcium-handling pathways in CKD-related cardiac injury (Li et al., 2026). Myokine- and EV-related signals may modulate these pathways. Among CKD studies, evidence is most developed for experimental EV interventions and for macrophage- or kidney-derived EV pathways, whereas direct evidence that endogenous skeletal muscle-derived mediators are dominant drivers of CKD-associated cardiac remodeling remains limited (Wang B. et al., 2019).

Cardiac fibroblasts and endothelial cells provide the main link between metabolic disturbance and structural remodeling. In cardiac fibroblasts, persistent inflammatory, oxidative, and TGF-β/Smad-related signaling promotes myofibroblast activation, connective tissue growth factor expression, collagen synthesis, and extracellular matrix accumulation (Travers et al., 2016). In endothelial cells and the myocardial microvasculature, oxidative stress and inflammatory activation reduce nitric oxide bioavailability, impair perfusion, and aggravate local hypoxia (Curaj et al., 2024; Edwards et al., 2026). These endothelial and microvascular abnormalities further restrict myocardial substrate oxidation and reinforce cardiomyocyte energetic stress.

Metabolic dysfunction and fibrosis should therefore not be viewed as separate downstream outcomes. Mitochondrial dysfunction and impaired substrate utilization increase oxidative stress and sensitize the myocardium to inflammatory and profibrotic signaling. In turn, extracellular matrix deposition and microvascular rarefaction impair oxygen diffusion and substrate delivery, further worsening metabolic inflexibility. This metabolic–fibrotic feed-forward loop provides a mechanistic explanation for the coexistence of myocardial metabolic reprogramming, fibrosis, and progressive cardiac dysfunction (Travers et al., 2016; Patel et al., 2022).

### 4.5 Bidirectional feedback and clinical correlates

Although current CKD evidence is stronger for skeletal muscle-to-heart signaling, reverse feedback from the stressed heart may also aggravate skeletal muscle dysfunction. Reduced cardiac output, impaired microvascular perfusion, sympathetic and renin–angiotensin–aldosterone system activation, and systemic inflammation can limit oxygen and nutrient delivery to skeletal muscle and promote catabolic signaling (Sabatino et al., 2021; Edwards et al., 2026). Evidence from heart failure and non-CKD cardiac injury models suggests that cardiac-derived myostatin may contribute to skeletal muscle wasting through ActRIIB–Smad2/3-dependent atrophy signaling (Breitbart et al., 2011). However, whether this cardiac-to-muscle endocrine feedback substantially contributes to CKD-associated sarcopenia remains insufficiently defined.

Human studies demonstrate associations between skeletal muscle phenotypes and cardiovascular structural or prognostic outcomes in CKD and dialysis populations. Higher skeletal muscle density or mass has been associated with lower thoracic aortic calcification burden and slower progression, whereas lower skeletal muscle density has been associated with greater cardiac valve-calcification burden (Li et al., 2025; Wang XX. et al., 2025). In cross-sectional indirect-effect analyses, skeletal muscle density statistically accounted for a small proportion of the associations between selected inflammatory or metabolic indices and thoracic aortic calcification (Wu et al., 2025); these estimates do not establish temporal sequence or biological mediation. Skeletal muscle density and muscle adipose-infiltration phenotypes have also been associated with mortality in dialysis cohorts (Sheng et al., 2023; Zheng et al., 2025). Exploratory human EV profiling has identified altered EV-associated miRNA patterns in CKD, but the human component was small and cross-sectional. The design characteristics and interpretive boundaries of these studies are summarized in **Table 2**. Collectively, the available human evidence supports skeletal muscle–cardiovascular phenotypic relationships rather than direct molecular skeletal muscle-to-heart causality.

Overall, CKD-associated skeletal muscle–heart communication involves upstream systemic stressors, circulating mediators, and cell-specific cardiac responses. Upstream uremic, inflammatory, oxidative, metabolic, hypoxic, and neurohumoral stressors reshape skeletal muscle and cardiac homeostasis. Intermediate myokine-, cytokine-, EV-, and miRNA-related signals transmit or amplify these disturbances across tissues. Downstream cardiac target cells convert these inputs into mitochondrial dysfunction, metabolic inflexibility, endothelial impairment, fibroblast activation, and fibrotic remodeling. Feedback from the stressed heart could further worsen skeletal muscle catabolism, closing the loop between sarcopenia and cardiac dysfunction.

## 5 Therapeutic opportunities

### 5.1 Exercise and rehabilitation-based interventions

Exercise is a physiological strategy for restoring adaptive skeletal muscle–heart communication (Chow et al., 2022; Shero et al., 2025). Unlike CKD-associated signaling, which is persistent and non-contraction-dependent, exercise-induced signaling is transient, contraction-coupled, and followed by recovery. Aerobic, resistance, and combined training may improve muscle oxidative capacity, endothelial function, autonomic balance, systemic inflammation, and cardiorespiratory reserve. Clinical practice guidelines support exercise and lifestyle interventions across CKD populations, with individualized adjustment according to frailty, comorbidity, dialysis status, and safety considerations (Baker et al., 2022). In hemodialysis patients, a randomized trial reported that a 6-month intradialytic cycling program reduced left ventricular mass and was safe, deliverable, and well tolerated (Graham-Brown et al., 2021). This trial provides human interventional evidence for improvement in a cardiac imaging phenotype but did not determine whether the reduction in left ventricular mass was mediated by skeletal muscle-derived myokines, EVs, or other inter-organ signals (**Table 2**). In CKD, exercise and rehabilitation may improve physical performance while shifting the signaling milieu toward lower inflammation, better mitochondrial function, and more adaptive myokine responses. Future trials should integrate functional outcomes with circulating myokine profiles, EV/miRNA signatures, cardiac imaging, and metabolic biomarkers to determine whether exercise truly modifies the skeletal muscle–heart axis.

### 5.2 Targeting myokine and inflammatory signaling

Myokine- and cytokine-related pathways provide potential pharmacological entry points. Inhibition of the myostatin/activin–ActRIIB pathway may improve muscle mass and strength (Rodgers and Ward, 2022; Shi et al., 2023). ActRII ligand inhibition has also been explored in experimental cardiac remodeling models (Castillero et al., 2020). However, whether this strategy can simultaneously improve skeletal muscle wasting and cardiac remodeling in CKD remains incompletely validated (Verzola et al., 2019). Similarly, modulation of chronic IL-6, TNF-α, or other inflammatory pathways may reduce catabolic and fibro-inflammatory signaling. Targeting IL-6-related inflammation has shown translational relevance in CKD-associated high-risk inflammatory settings (Ridker et al., 2021). In the RESCUE trial, ziltivekimab reduced inflammatory and thrombotic biomarkers in patients with CKD and systemic inflammation; however, the study did not assess skeletal muscle phenotypes, muscle-derived IL-6, cardiac remodeling, or skeletal muscle–heart communication (**Table 2**). However, broad immunosuppression carries safety concerns in CKD. Protective myokines such as irisin, apelin, musclin, and FSTL1 may also represent candidate pathways, but their relevance in CKD remains insufficiently validated. Therapeutic studies should test tissue selectivity and require benefit in both skeletal muscle and the heart, rather than relying on circulating mediator changes alone.

### 5.3 EV and miRNA-based therapeutic strategies

EV and miRNA-based approaches are attractive because they may allow cargo-based modulation of inter-organ communication. The miR-26a restoration model suggests that engineered exosomes can improve insulin signaling, reduce skeletal muscle atrophy, attenuate cardiac fibrosis, and improve cardiac function in CKD-related settings (Wang B. et al., 2019). Other miRNAs, including miR-29 family members, have been implicated in muscle atrophy and kidney fibrosis models (Wang H. et al., 2019; Wang et al., 2020a). Nevertheless, EV-based therapies face unresolved challenges in source specificity, cargo heterogeneity, biodistribution, off-target effects, immune compatibility, scalable manufacturing, long-term safety, and reliable potency assays (Grange and Bussolati, 2022; Ng et al., 2025). Importantly, therapeutic EV delivery should not be interpreted as evidence that endogenous EVs from the same source mediate disease progression. Therapeutic gain-of-function and endogenous pathological signaling are related but distinct concepts.

### 5.4 Integrated multimodal strategies

Because CKD-associated skeletal muscle–heart crosstalk arises from multiple upstream drivers, single-target interventions may be insufficient (House et al., 2019; Edwards et al., 2026). Integrated strategies combining exercise rehabilitation, nutritional support, correction of metabolic acidosis, anti-inflammatory or antioxidant approaches, dialysis optimization, anemia management, and cardiovascular risk control may be required to improve both skeletal muscle and cardiovascular outcomes. These strategies should align with contemporary CKD care frameworks and be individualized according to kidney function, comorbidity burden, frailty, and cardiovascular risk (Kidney Disease: Improving Global Outcomes (KDIGO) CKD Work Group, 2024). Biomarker-guided stratification may further identify patients with predominant muscle wasting, inflammatory activation, EV/miRNA dysregulation, or cardiac metabolic dysfunction. Future intervention studies should evaluate skeletal muscle function, circulating mediators, cardiac imaging, and clinical outcomes within the same protocol.

## 6 Limitations and future validation

Several limitations currently restrict interpretation of skeletal muscle–heart crosstalk in CKD. First, evidence remains fragmented. Many studies examine a single mediator, one organ, or one disease model, but few establish the complete causal chain from source tissue to mediator release, circulation, cardiac target-cell uptake, molecular target engagement, and organ-level phenotype rescue. Without this sequence, changes in circulating mediators should be interpreted as associations rather than definitive inter-organ communication. Because interpretation of the human evidence depends strongly on study design, population characteristics, clinical context, follow-up, analytical adjustment, outcome selection, and generalizability, these features are summarized in **Table 2**.

Second, directionality remains incompletely established. Current CKD evidence is more extensive for skeletal muscle-to-heart signaling than for cardiac-to-muscle feedback. However, CKD affects the kidney, immune system, vasculature, skeletal muscle, and myocardium simultaneously. Shared systemic stress may therefore generate parallel abnormalities in multiple organs without requiring direct molecular transfer between skeletal muscle and the heart. Future studies should use tissue-specific genetic models, organ-selective gain- or loss-of-function approaches, and rescue experiments to distinguish causal inter-organ signaling from shared pathway activation.

Third, EV source attribution is a major unresolved problem. Circulating EVs in CKD may originate from the kidney, immune cells, platelets, endothelium, skeletal muscle, myocardium, and other tissues. Bulk plasma EV profiling cannot determine whether a specific EV cargo is skeletal muscle-derived or whether it directly targets cardiac cells. Future work should incorporate source-specific EV labeling, single-EV analysis, cell-type-resolved cargo profiling, and *in vivo* tracking strategies to define the origin, biodistribution, and target-cell uptake of EV subpopulations.

Fourth, CKD heterogeneity must be considered. CKD stage, dialysis modality, diabetes, aging, malnutrition, physical inactivity, inflammation, mineral-bone disorder, anemia, and cardiovascular comorbidity may all reshape skeletal muscle and myocardial responses. These factors may alter both mediator production and cardiac responsiveness. Therefore, findings from one CKD model or patient subgroup may not generalize across the full CKD spectrum.

Future studies should combine longitudinal human cohorts with mechanistic animal and cellular models. Single-cell and single-nucleus RNA sequencing, spatial transcriptomics, proteomics, metabolomics, stable-isotope metabolic flux analysis, and advanced cardiac imaging may help define how skeletal muscle, kidney, immune, vascular, and cardiac compartments interact over time. Human exercise intervention studies should integrate muscle function, body composition, circulating myokines, EV cargoes, cardiac structure, myocardial strain, and clinical outcomes. These approaches will determine whether the skeletal muscle–heart axis is useful for biomarker discovery, patient stratification, or therapy in CKD.

## 7 Conclusions and future perspectives

In summary, CKD may convert physiological skeletal muscle–heart communication into a maladaptive and partly bidirectional axis. Uremic toxin retention, chronic inflammation, oxidative stress, hypoxia, metabolic disturbance, and neurohumoral activation alter both skeletal muscle and myocardial homeostasis. Through changes in myokines, EV cargoes, and miRNA-mediated signaling, these systemic disturbances may contribute to cardiomyocyte metabolic inflexibility, endothelial dysfunction, fibroblast activation, extracellular matrix deposition, and progressive cardiac remodeling. Conversely, cardiac-derived endocrine factors, impaired peripheral perfusion, and persistent neurohumoral activation may further promote skeletal muscle catabolism.

Although this framework helps integrate CKD-associated sarcopenia and cardiovascular remodeling, direct causal evidence remains incomplete. In particular, studies that simultaneously identify the source tissue, molecular mediator, cardiac target cell, and organ-level phenotype remain limited, and cardiac-to-muscle feedback in CKD requires further validation. Future work combining longitudinal human cohorts, mechanistic animal models, cell-specific omics, EV-tracing technologies, and interventional studies will be essential to determine whether this axis can support biomarker development, patient stratification, or therapeutic targeting in CKD.

## Glossary

ActRIIA: activin receptor type IIA
ActRIIB: activin receptor type IIB
AKT: protein kinase B
AMPK: AMP-activated protein kinase
ANGPTL4: angiopoietin-like protein 4
ANP: atrial natriuretic peptide
APJ: apelin receptor
Bax: Bcl-2-associated X protein
Bcl-2: B-cell lymphoma 2
BDNF: brain-derived neurotrophic factor
BNP: B-type natriuretic peptide
CF: cardiac fibroblasts
cGMP: cyclic guanosine monophosphate
CKD: chronic kidney disease
CM: cardiomyocytes
CNP: C-type natriuretic peptide
DIP2A: disco-interacting protein 2 homolog A
EC: endothelial cells
eNOS: endothelial nitric oxide synthase
ERK: extracellular signal-regulated kinase
EVs: extracellular vesicles
FNDC5: fibronectin type III domain-containing protein 5
FoxO1: forkhead box O1
FST: follistatin
FSTL1: follistatin-like protein 1
GLUT4: glucose transporter type 4
gp130: glycoprotein 130
HDAC4: histone deacetylase 4
HFpEF: heart failure with preserved ejection fraction
HIIT: high-intensity interval training
IGF-1: insulin-like growth factor 1
IGF-1R: insulin-like growth factor 1 receptor
IL-6: interleukin-6
IL-6R: interleukin-6 receptor
IL-7: interleukin-7
IL-10: interleukin-10
IL-15: interleukin-15
Imm: immune cells
JAK: Janus kinase
MAPK: mitogen-activated protein kinase
METRNL: meteorin-like protein
miR-1: microRNA-1
miR-21: microRNA-21
miR-26a: microRNA-26a
miR-133a/b: microRNA-133a/b
miR-155: microRNA-155
miRNAs: microRNAs
mRNA: messenger RNA
mTOR: mechanistic target of rapamycin
MuRF1: muscle RING-finger protein 1
NP: natriuretic peptide
NPR-C: natriuretic peptide clearance receptor
OSTN: osteocrin
PGC-1α: peroxisome proliferator-activated receptor gamma coactivator 1-alpha
PI3K: phosphoinositide 3-kinase
PKA: protein kinase A
PKG: protein kinase G
PTEN: phosphatase and tensin homolog
SMD: skeletal muscle density
Smad2/3: SMAD family member 2/3
sTNF-R: soluble tumor necrosis factor receptors
TGF-β: transforming growth factor-beta
TNF-α: tumor necrosis factor-alpha
TrkB: tropomyosin receptor kinase B
UCPs: uncoupling proteins
UTR: untranslated region
VSMC: vascular smooth muscle cells
GC-A: guanylyl cyclase-A
NPRA: natriuretic peptide receptor A
RAAS: renin–angiotensin–aldosterone system.
